# Audit of short term outcomes of surgical and medical second trimester termination of pregnancy

**DOI:** 10.1186/1742-4755-6-16

**Published:** 2009-09-30

**Authors:** Annamarie Mauelshagen, Lynn C Sadler, Helen Roberts, Mahesh Harilall, Cynthia M Farquhar

**Affiliations:** 1 Department of Obstetrics and Gynaecology, University of Auckland, Auckland, New Zealand; 2 National Women's Health, Auckland District Health Board, Auckland, New Zealand

## Abstract

**Background:**

As comparisons of modern medical and surgical second trimester termination of pregnancy (TOP) are limited, and the optimum method of termination is still debated, an audit of second trimester TOP was undertaken, with the objective of comparing the outcomes of modern medical and surgical methods.

**Methods:**

All cases of medical and surgical TOP between the gestations of 13 and 20 weeks from 1st January 2007 to 30th June 2008, among women residing in the local health board district, a tertiary teaching hospital in an urban setting, were identified by a search of ICD-10 procedure codes (surgical terminations) and from a ward database (medical terminations). Retrospective review of case notes was undertaken. A total of 184 cases, 51 medical and 133 surgical TOP, were identified. Frequency data were compared using Chi-squared or Fischer's Exact tests as appropriate and continuous data are presented as mean and standard deviation if normally distributed or median and interquartile range if non-parametric.

**Results:**

Eighty-one percent of surgical terminations occurred between 13 to 16 weeks gestation, while 74% of medical terminations were performed between 17 to 20 weeks gestation. The earlier surgical TOP occurred in younger women and were more often indicated for maternal mental health. Sixteen percent of medical TOP required surgical delivery of the placenta. Evacuation of retained products was required more often after medical TOP (10%) than after surgical TOP (1%). Other serious complications were rare.

**Conclusion:**

Both medical and surgical TOP are safe and effective for second trimester termination. Medical TOP tend to be performed at later gestations and are associated with a greater likelihood of manual removal of the placenta and delayed return to theatre for retained products. This case series does not address long term complications.

## Background

Termination of pregnancy (TOP) is the most common procedure performed worldwide and each year in New Zealand approximately 18,000 TOPS are performed [1,2]. Both medical and surgical termination techniques have evolved over the last 30 years, and are continually improving with regard to effectiveness, acceptability and rate of complications [1].

For the past 50 years the procedure has been completed using surgical approaches, mostly dilatation and evacuation (D&E) of the uterus. However, over the past two decades the development of medical approaches using antiprogesteronic agents and prostaglandin analogues has provided an alternative for women seeking TOP [3]. Medical TOP involves oral mifepristone, a progesterone antagonist which sensitises the myometrium to prostaglandin induced contractions and ripens the cervix, followed by misoprostol, a synthetic prostaglandin E1 analogue, orally and/or vaginally, which causes uterine contractions and cervical dilatation [2,3]. The addition of the progesterone antagonist reduces the induction to abortion interval, analgesia required and prostaglandin dose [3,4].

Surgical termination consists of prior dilapan rod insertion and misoprostol priming to dilate and soften the cervix, followed by D&E under local or general anaesthetic [3]. Dilapan rods are hydrophilic polymer rods which work by absorbing fluid and slowly expanding, therefore causing cervical dilatation [4].

Ideally, TOP should be performed in the first trimester as this is safer and more acceptable. However, for some women, second trimester TOP is unavoidable; screening for Trisomy 21 does not occur until late in the 1st trimester, there may be poor access to services or fear of disclosure of the pregnancy [3,5]. While second trimester TOP accounts for 10-15% of termination worldwide [6,7], it accounts for two-thirds of all major termination related complications [7,8].

Comparisons of modern medical and surgical methods for second trimester TOP are limited, and the optimum method of termination is debated. Only one study of women undergoing second trimester TOP, comparing D&E to mifepristone and misoprostol, met the criteria for a Cochrane Review in 2008 [3,9]. This study included only 18 women and there was no evidence of a difference in outcomes with the exception of adverse events and overnight stay, which were more common in the medical group [9]. Further studies were suggested.

The objective of this study was to audit second trimester TOP at National Women's Hospital, Auckland, New Zealand, over an eighteen month period by retrospective review of case notes. Specific aims were to compare patient characteristics and complication rates of modern medical and surgical TOP methods.

## Materials and methods

Cases include all women residing in the Auckland District Health Board area who underwent modern medical or surgical second trimester TOP (13 to 20 weeks) at National Women's Health, Auckland New Zealand, from January 2007 to June 2008. All medical and surgical second trimester TOPs are performed at the ADHB. No other facilities are licensed to perform second trimester TOP in the Auckland region. Women who meet the criteria for TOP between 13 and 20 weeks gestation have a consultation with a certifying consultant for TOP and are offered either medical or surgical TOP. The advantages and disadvantages of both methods are discussed including the benefits of detailed post mortems where fetal abnormality is present. Cases were identified from ICD-10 procedure codes for surgical TOP and from a database maintained by the service for medical TOP. Coding data were searched for presence of any further medical TOPs but no other cross-verification was possible. Patient characteristics and clinical details were obtained from the electronic case notes by retrospective review.

The protocol used for medical TOP was as follows: 600 mg mifepristone orally, admission to gynaecology ward 36-48 hours later, 800 micrograms misoprostol vaginally then 400 micrograms misoprostol orally at three hourly intervals, with a maximum of four oral doses. If significant bleeding occurred prior to planned admission, admission occurred at that time and the misoprostol regimen commenced. Manual removal of the placenta was recommended after delivery if the placenta did not deliver spontaneously. Pain relief was offered at regular intervals during the procedure according to a protocol that included morphine or pethidine given intravenously. In women with more severe pain a patient controlled anaesthesia pump (PCA) was made available that administered morphine.

The protocol for surgical termination was as follows: dilapan rod insertion 12 to 24 hours prior to the procedure, misoprostol 400 micrograms orally or vaginally one to three hours before the procedure, followed by D&E [2,3]. During the procedure intravenous fentanyl (maximum dose of 20 mg) and midazolam (maximum dose 3 mg) was given and rectal suppositories of paracetamol (1 gm) and voltaren (75 mg) were given.

The following information was extracted from medical records: age, parity, ethnicity, history of previous TOP, previous caesarean section, indication for TOP, contraception when pregnancy resulted from contraceptive failure, gestation at TOP, complications and follow up arrangements recorded. Additional information collected for patients who underwent medical TOP included misoprostol dose, time from mifepristone to delivery, time from admission to delivery, time from delivery of the fetus to delivery of the placenta, whether manual removal of the placenta was required, and nights spent in hospital. Additional information for patients who underwent surgical TOP included blood loss, number of dilapan rods, anaesthetic method and length of procedure.

The following were regarded as complications of the procedure:

• haemorrhage requiring transfusion;

• infection requiring intravenous antibiotics;

• retained products of conception requiring dilatation and curettage;

• laparotomy (any indication including repair of uterine rupture, hysterectomy);

• uterine perforation and cervical laceration; and

• unscheduled re-admission. Unscheduled re-admission was defined as a hospital stay of more than three hours.

### Statistical methods

Statistical analyses were performed using STATA9 software. Statistical significance was defined as p < 0.05. Frequency data were compared using Chi-squared or Fischer's Exact tests as appropriate. Continuous data are presented as mean and standard deviation if normally distributed or median and interquartile range if non-parametric.

## Results

A total of 184 terminations of pregnancy, between 1st January 2007 and 30th June 2008 at 13 weeks to 19 weeks and 6 days gestation, were identified. All case notes were available for review. Fifty-one were medical TOP and 133 were surgical. Two women had two TOP during the time period. Both episodes are included in the dataset.

There was a significant difference in gestational age between the two methods, with 81% of surgical TOP performed between 13 and 16 weeks, and 74% of medical TOP were performed between 17 and 20 weeks.

Surgical TOP was more common among younger women, having TOP at earlier gestations, and most commonly for maternal mental or physical health reasons (table 1).

**Table 1:** Demographic and clinical details of women undergoing mid-trimester TOP by method of termination

	**Medical** ***n *= 51**		**Surgical** ***n *= 133**		**P**
	**n**	**%**	**n**	**%**	
**Age (yrs)**					
<20	4	8	32	24	
20-24	12	24	37	28	
25-29	6	12	22	16	
30-34	11	22	16	12	
35-39	12	24	16	12	
≥40	6	12	10	7	0.04
Median (IQR)	31	(24-38)	24	(20-32)	<0.001
**Ethnicity**					
European	19	37	47	35	
Maori	4	8	17	13	
Pacific	12	24	35	26	
Asian	14	27	28	21	
Other	2	4	5	4	
Unknown	0		1	1	0.9
**Parity**					
Nulliparous	22	43	71	53	
Parous	25	49	53	40	
Unknown	4	8	9	7	0.5
**Previous caesarean section**					
Yes	7	14	15	11	
No	35	69	107	80	
Unknown	9	18	11	8	0.2
**Previous TOP**					
No	31	61	88	66	
Yes	16	31	36	27	
Unknown	4	8	9	7	0.8
**Indication for TOP**					
Maternal mental or physical health	41^1^	80	133^2^	100	
Intrauterine death	5	10	0	0	
Spontaneous rupture of membranes	5	10	0	0	
**Gestation (wks)**					
13	0		6	5	
14	4	8	35	26	
15	3	6	31	23	
16	6	12	36	27	
17	8	16	23	17	
18	14	27	1	1	
19	14	27	1	1	
20	2	4	0		<0.001
Median (IQR)	18	(16-19)	15	(14-16)	<0.001

^1 ^Includes two TOP for maternal physical health and 21 TOPs for adjustment disorder with depressed mood following a diagnosis of fetal abnormality

^2 ^Includes 114 TOPs for adjustment disorder with depressed mood following a diagnosis of fetal abnormality

All ten TOP following intrauterine fetal demise and spontaneous rupture of the membranes had medical induction of labour. Where the indication for termination was "adjustment disorder and depressed mood following a diagnosis of a fetal abnormality" medical TOP was the preferred method.

Overall 30% of women undergoing mid-trimester TOP had a history of prior TOP, and 10% had two or more prior TOP. Pregnancy following contraceptive failure occurred in at least 73 cases (40%). Among the 73 of women who had conceived following failed contraception, 27% underwent medical TOP and 44% underwent surgical TOP.

Data were unavailable for a further 21%. The most common method of contraception among contraceptive failures leading to TOP was condom failure when condoms were used alone (66%), followed by the combined oral contraceptive pill (COCP) (14%), the emergency contraceptive pill (ECP) (4%), the progesterone only pill (3%), Depo Provera (2%), condoms in combination with the COCP (1%), condoms in combination with the ECP (4%) and unknown in 3%.

Within the medical TOP group seven women (14%) aborted following mifepristone and the initial vaginal dose of misoprostol. These included two women who had spontaneous rupture of membranes (SROM), and one with intra-uterine fetal demise (IUFD). Two women who had an IUFD did not require misoprostol. The remaining women aborted successfully following 1-4 doses of oral misoprostol. Only 10% of women required 4 oral doses. Twenty-one (41%) of women were managed as a day case and a further 43% had one over-night stay. Table 2 reports on the time involved in medical TOP. The median hospital stay, from admission for misoprostol to discharge, was 24 hours. The median length of labour was 5.1 hours (3.3-9.5 interquartile range) among the 70% of women for whom this data were available. Induction-to-abortion interval, defined as the time interval from first misoprostol administration to delivery of fetus, was 8 hours (5-11 interquartile range). Eight (16%) of women went to operating theatre for manual removal of the placenta. The time from delivery to manual removal of the placenta was less than 5 hours in 6 women and the remaining two were within 10 hours and included one woman with a partial delivery of the placenta.

**Table 2:** Details of medical terminations (13-19 weeks)

	**Medical TOP** **n = 51**	
	**n**	**IQRe**

Median dose of misoprostol given (total)	2	0-4
Median hours from mifipristone dose to delivery of fetus	53	49-56
Median hours from misoprostol to delivery of fetus	8	5-11
Median hours from admission to discharge	24	10-30
Median hours from delivery of fetus to delivery of placenta	0.4	0-2

Data are median (interquartile range)

In almost all cases where the number of dilapan rods used was known, two were inserted into the cervix. Two women, at 13 and 14 weeks gestation did not have dilapan rods inserted prior to D&E. Eighty percent of surgical TOP were carried out under local anaesthetic with sedation. The mean length of procedure was 8.2 minutes (standard deviation 2.1), and mean blood loss 199 ml (standard deviation 97).

A comparison of complications by method is shown in Table 3 Women undergoing medical TOP were at a significantly higher risk of requiring evacuation of retained products of conception (10% among medical TOP compared to 1% of surgical TOP; p= 0.02). One woman, with a history of postpartum haemorrhage, in the medical TOP group, required a three unit blood transfusion for excessive bleeding post delivery.

**Table 3:** Complications following termination of pregnancy (13-19 weeks) by method of termination

	**Medical** ***n *= 51**		**Surgical** ***n *= 133**^**1**^		**P**
	**n**	**%**	**n**	**%**	
**Nights in hospital not including readmissions**					
0	21	41	**-**		
1	22	43			
2	6	12			
3	1	2			
5	1	2			
					
**Manual Removal of placenta**^**2,3**^	8	16	**-**		

					

**Complications**					
Readmission > 3 hours	3	6	6	4	0.7
Evacuation for retained products of conception^2^	5	10	2	1	0.02
Endometritis requiring intravenous antibiotics	1	2	3	2	1.0
Blood transfusion	1	2	0	0	0.3

^1 ^no surgical cases were admitted

^2 ^manual removal of placenta and evacuation for retained products of conception were not mutually exclusive

^3 ^there was one case of partial expulsion of the placenta but required manual removal at 17 hours.

Sixty-one percent of women had been prescribed contraception or had a plan documented prior to discharge. Among the 73 who conceived following contraceptive failure only 8 women (11%) did not have the plan for contraception recorded in their records.

## Discussion

This paper describes the outcomes of 133 surgical and 51 medical TOP between 13 and 20 weeks from January 1 2007 to June 30 2008 at one tertiary institution. Surgical termination is most often performed at an earlier gestation, with ninety-nine percent performed before the end of the 17th week, while medical TOP is almost always performed at or beyond 16 weeks. These differences reflect the ease and safety of early D&E; and the relative ease and safety of late medical induction. The decision about the method of second trimester TOP is made jointly between the women and the medical practitioner. All women are offered medical TOPs. This study has not explored the reasons for the decision and future assessment of this is planned. The difference in gestation between the two techniques makes comparing complications difficult. All medical TOPS were successful although 16% of cases required manual removal of the placenta. All other serious adverse events were comparable between the two groups, although a study of this size has a small chance of picking up a difference in rates of rare outcomes.

One explanation for the higher rates of evacuation for retained products of conception could be the higher gestations in the medical TOP group. Later gestation at the time of the TOP is associated with an increased risk of retained products although Autry et al [10] adjusted for gestation and still found an increase in risk following medical TOP. The outcomes reported by Autry are not strictly comparable as the mean gestation for medical treatment is 20 weeks and the medical method is not clearly described (a combination of laminaria and misoprostol) [10]. Autry also reported that the use of misoprostol was associated with fewer complications (failed initial procedure and retained products) than laminaria for medical terminations [10]. The percentage of women requiring manual removal of placenta following medical termination of pregnancy in our study (16%), is also high compared to other studies using similar techniques [5,11]. Ashok [5] reported 7% requiring manual removal of placenta, and Goh 10 found an incidence of 5%. Both of these studies had a significantly larger subject group than this study, with 999 and 386 subjects respectively.

Only one woman required transfusion following medical TOP. None of the surgical patients required a blood transfusion which compares favourable with previous studies on D&E [12]. The large proportion of procedures carried out under LA (80%) may explain this outcome, as use of GA has been associated with increased blood loss [12].

These findings are consistent with previous reported series [5,10] and the one small published randomised controlled trial [5]. Even in the larger series, there is no evidence of increased complications among either surgical or medical TOP other than the increased rate of evacuation for retained products following medical TOP.

Other findings from this report have highlighted some deficiencies in recording contraception at the time of discharge. It is unknown if this reflects poor record keeping and this will be addressed by the service in the future. For those women who had failed contraception leading to the TOP, only 11% did not have a contraceptive plan.

There are several limitations to this study. Firstly, this is a retrospective review of complications. Ideally, clinical data collection should be prospective. Secondly, ascertainment of complications may not have been complete in this study as identification of major complications was reliant on re-referral or re-admission to the hospital were the procedure was performed. Thirdly, the study groups are not strictly comparable because of the difference in gestation and indications. Finally, the study does not address long term adverse effects such as increased risk of preterm birth and sub-fertility from either second trimester medical or surgical TOPs.

Previous studies would suggest that even at the upper limits of mid-trimester, D&E is associated with fewer complications, shorter duration, and less pain and emotional distress [5,9,11]. To undertake D&E safely and effectively in the middle trimester of pregnancy, specialist training and an adequate caseload are required, and the Royal College of Obstetricians and Gynaecologists (RCOG) and the World Health Organisation (WHO) advise inexperienced professionals to use medical methods [13,14]. What is not clear from the published literature is whether there is any long term risk resulting from late TOP. This question might be addressed in a randomised trial, though in the USA poor recruitment made such a study unfeasible [9]. Our preliminary audit raises a number of questions regarding the outcomes and the optimal method for second trimester TOP. Further research should consider an experimental study design such as a randomised controlled trial comparing D&E with mifepristone and misoprostol in the 15th to 18th weeks of pregnancy for TOP including an assessment of patient satisfaction and long term outcomes. A case-control study might also be of value in addressing some of the less common adverse outcomes.

## Competing interests

The authors declare that they have no competing interests.

## Authors' contributions

AM participated in the study planning, identifying the patients and extracting the information from the patient's files and writing an initial draft of the manuscript. LS participated in the study planning, identifying the patients, analyzing the data and preparing the manuscript. HR participated in the study planning and preparing the manuscript with particular reference to the contraceptive aspects of the study. MH participated in the study planning, analyzing the data and preparing the manuscript. CF participated in the study planning, identifying the patients, analyzing the data and preparing the manuscript.
